# Measuring Managerial, Collegial, and Organizational Aspects Associated With Digital Health Competence in Healthcare Professionals: Validation of the Chinese Version of the DigiComInf Instrument

**DOI:** 10.1155/jonm/8854459

**Published:** 2025-06-16

**Authors:** Lu Gao, Jinni Wang, Meilian Chen, Jingxin Wei, Xiaoyan Liao

**Affiliations:** ^1^School of Nursing, Southern Medical University, Guangzhou, China; ^2^School of Nursing, Guangzhou Medical University, Guangzhou, China

**Keywords:** colleague, digital health competence, healthcare professionals, influence, management, organization, validation

## Abstract

**Background:** Managerial, collegial, and organizational influences have been recognized as critical factors for developing digital health competence among healthcare professionals, but there is currently a lack of validated Chinese instruments to evaluate these factors.

**Aims:** To culturally adapt and validate the Chinese version of the DigiComInf (aspects associated with digital health competence) instrument among Chinese healthcare professionals.

**Method:** The DigiComInf instrument was translated into Chinese following the established guidelines. The cultural adaptation involved expert review and cognitive interviews. A total of 311 healthcare professionals were sampled to test reliability and validity of the instrument, with 30 participants undergoing a retest after a 2-week interval. Item analysis, internal consistency, test-retest reliability, content validity, convergent validity, discriminant validity, and construct validity were examined.

**Results:** Item analysis indicated satisfactory item discrimination (critical values range: 17.63–26.70), item correlation (corrected item-total correlation coefficient > 0.4), and item homogeneity. Internal consistency (Cronbach's α = 0.96) and test-retest reliability (Intraclass correlation coefficient = 0.96, 95% CI 0.91–0.98) were excellent. The scale content validity index was excellent (0.97). Principal component analysis extracted three common factors, explaining 80.54% of the cumulative variance. Confirmatory factor analysis confirmed a well-fit 3-factor model (*χ*^2^/*df* = 3.19, CFI = 0.94, NFI = 0.92, TLI = 0.93, IFI = 0.94, RMSEA = 0.10, and SRMSR = 0.04), with acceptable convergent and discriminant validity.

**Conclusion:** The Chinese version of the DigiComInf is a reliable and valid instrument for assessing managerial, collegial, and organizational influences on the development of digital health competence among Chinese healthcare professionals.

**Implications for Nursing Management:** This study presents a validated instrument to evaluate managerial, collegial, and organizational influences on healthcare professionals' digital health competence. Healthcare managers, healthcare providers, researchers, and policymakers can use it to accurately identify modifiable socio-organizational factors associated with digital health competence among various healthcare professionals in various settings, thereby informing targeted interventions to enhance their digital health competence.

## 1. Introduction

Digital health has emerged as a significant trend in global healthcare services. The ongoing digital transformation in this sector is redefining the roles and responsibilities of healthcare professionals [1, 2], highlighting an urgent need for digital health competence. According to the World Health Organization's 2013 guidelines, healthcare professionals encompass doctors, nurses, midwives, dentists, pharmacists, and other personnel [3]. This competence is increasingly recognized as one of the core competencies, enabling these professionals to design digital solutions, assess their impact on patient care, and determine the best way for implementation in their practice [4, 5]. Studies indicate that inadequate digital health competence may negatively impact healthcare professionals' acceptance and utilization of digital technology and compromise patient safety [6, 7]. Therefore, systematic strategies to enhance digital health competence have become imperative for delivering high-quality digital healthcare services.

Demographic factors, such as younger age, higher level of education, and recent graduation, are associated with higher information literacy [8, 9]. However, these factors are difficult to modify. Resistance to change, inadequate infrastructure, lack of institutional support, and insufficient training are identified as the primary barriers to successful implementation of digital solutions [6, 9, 10]. These barriers reflect organizational behavioral complexities rather than mere technical challenges. Three modifiable factors emerge as critical targets for developing digital health competence among healthcare professionals: support from management, organizational practices, and collegial influence [11]. Managers, as resource allocators, decision-makers, and facilitators, are vital in delivering digital healthcare. Their perceptions and acceptance of digital solutions establish foundational conditions for healthcare professionals' competence development [12], with leadership behavior positively or negatively influencing staff development of digital health competence [13]. Nursing professionals underscore managers' responsibility to uphold digital proficiency standards during workforce transitions and technological upgrades [14]. Additionally, colleagues' social influence impacts individuals [15], as their acceptance and competence levels affect others' willingness to adopt digital solutions in their work [11]. Organizational training and practice significantly affect the development of healthcare professionals' digital health competence. Appropriate training improves nurses' informatics competence and reduces the likelihood of work-related errors [16]. Critically, culturally adapted assessment instruments enable precise identification of modifiable determinants, thereby informing targeted interventions to enhance digital health competence in settings that optimize digital health service delivery.

Existing assessment instruments predominantly address digital health literacy [17], digital application skill [18], and digital health competence [19]. However, instruments to evaluate the influencing factors of digital health competence in healthcare professionals are rare. The DigiComInf (aspects associated with digital health competence) instrument addresses this gap by quantifying managerial, collegial, and organizational influences on healthcare professionals' digital health competence. Originally validated in Finnish with 817 healthcare professionals across 9 institutions [11, 20], its cross-cultural adaptability has prompted translation into 15 languages for an ongoing large-scale international cross-sectional study on the digital health competence of healthcare professionals. As participants in this international initiative, this study aimed to establish the psychometric properties of the Chinese version of the DigiComInf instrument among Chinese healthcare professionals.

## 2. Methods

### 2.1. Study Design

A cross-sectional study was conducted that adhered to the strengthening the reporting of observational studies in epidemiology (STROBE) guidelines.

### 2.2. Participants

Participants were recruited via professional social media networks and onsite methods by using convenience sampling. QR-coded recruitment posters were deployed on professional social media networks. In addition, healthcare professionals present at local academic conferences and continuing education workshops were invited to participate. The inclusion criteria of participants were as follows: (1) healthcare professionals working in healthcare organizations, (2) with at least 1 year of work experience, and (3) consent to participate. The exclusion criteria were as follows: (1) individuals who retired from work, (2) healthcare students, (3) participants who completed the survey in under 150 s, and (4) those exhibiting erratic response patterns, such as inconsistent answers, extreme scores, or geometric click patterns. According to the World Health Organization's 2013 guidelines, healthcare professionals include doctors, nurses, midwives, dentists, pharmacists, and other personnel.

A minimum sample size of 200 is recommended for confirmatory factor analysis (CFA) [21]. Finally, a total of 311 participants were included in the study, which were randomly divided into two groups: Group A (*n* = 101) for principal component analysis (PCA) and Group B (*n* = 210) for CFA. To examine the test-retest reliability of the instrument, 30 participants completed the questionnaire again after a 2-week interval.

### 2.3. The Instrument

The DigiComInf instrument consisted of three domains, comprising a total of 15 items: support from management (6 items), organizational practices as part of digital competence development (4 items), and colleagues' adoption and influence (5 items). Each item was rated on a four-point Likert scale: 1 = completely disagree; 2 = partially disagree; 3 = partially agree; and 4 = completely agree. The total score ranges from 0 to 60, with a higher score indicating a higher level of support. For each domain, a mean value of ≤ 2.49 indicates a low level, 2.50 to 3.49 indicate an intermediate level, and ≥ 3.50 indicates a high level [11]. The DigiComInf instrument has been validated among healthcare professionals in tertiary, primary, and private healthcare settings (*n* = 817), demonstrating satisfactory internal consistency (Cronbach's α 0.74–0.88) and content validity (item content validity index (I-CVI) 0.75–1.00; scale CVI/average (S-CVI/Ave) 0.95) [20].

### 2.4. Translation and Cultural Adaption

**Figure 1:**
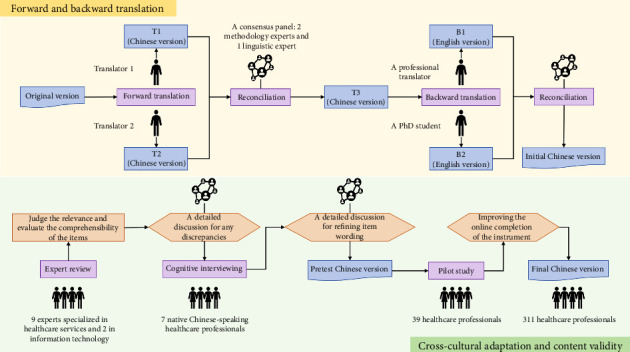
The translation and cultural adaptation process.

After authorization from the original developers, the DigiComInf instrument was translated into Chinese. In order to ensure a high-quality Chinese translation of the DigiComInf instrument, we followed a rigorous translation and cross-cultural adaptation process [22].  Figure 1 illustrates the translation and cross-cultural adaptation process.

#### 2.4.1. Forward and Backward Translation

Two bilingual nursing professionals (a nursing expert and a nursing master's degree student), who are native Chinese speakers, independently translated the English version of the DigiComInf instrument into Chinese. This process resulted in two forward translations (T1 and T2). A consensus panel evaluated and reconciled these versions, focusing on conceptual equivalence, clarity, and comprehensibility, producing a third version (T3). Next, the T3 version underwent back-translation into English by a professional translator with test for English majors-band 8 (TEM-8) qualification and a nursing PhD student. They were not aware of the original English versions and were knowledgeable about both cultures. The consensus panel (2 nursing researchers experienced in instrument development and a linguistic expert) reviewed and resolved any discrepancies between the back-translations and the original version, finalizing the initial Chinese version.

#### 2.4.2. Expert Review

To ensure content validity of the Chinese version of the DigiComInf instrument, a panel of 11 experts evaluated the relevance of its domains and the items using a 4-point ordinal scale (1 = not relevant to 4 = very relevant). This expert panel consisted of 9 healthcare specialists and 2 IT professionals. Additionally, the experts evaluated the comprehensibility of the items. The review experts worked independently of the research team. Potential discrepancies were systematically resolved through in-depth discussions by the consensus panel, which had been part of the translation process. The expertise and demographic background of the experts were shown in Table 1.

#### 2.4.3. Cognitive Interviewing

Cognitive interviewing, an established methodology for improving and refining questionnaire items [23], was utilized to evaluate the clarity and cultural appropriateness of the initial Chinese version. Common probes included questions, such as “Can you rephrase the item in your own words?” and “How did you decide on that answer?” Seven native Chinese speakers representing diverse professional backgrounds (physicians: *n* = 2; nursing professionals: *n* = 4; and health information technology technician: *n* = 1) were enrolled through purposive sampling. All participants received comprehensive briefing regarding study objectives and methodologies, with written informed consent obtained prior to the interviews. Demographic characteristics of cognitive interviewees were shown in Table 1.

The first author, trained in qualitative research methods, conducted cognitive interviews in a meeting room. Participants independently completed the Chinese version of the DigiComInf instrument and engaged in cognitive interviews. Field notes were taken. Linguistic modifications were deemed necessary if at least one participant (1) found an item difficult to understand, (2) showed mostly or completely inaccurate comprehension of an item, or (3) provided feedback indicating needed improvements, particularly regarding cultural relevance. The consensus panel determined whether to retain or alter item wording following expert review and cognitive interviewing. Any discrepancies affecting item clarity were resolved to create the pretest version of the Chinese DigiComInf instrument.

#### 2.4.4. Pilot Study

Thirty-nine healthcare professionals from a general hospital in Guangzhou, China, were selected for a pilot study conducted between April and May 2023. Participants' comments and feedback were used to improve the online administration of the instrument and to refine item formulations. As a result, the Chinese version of the DigiComInf instrument was created (Supporting Information 1).

### 2.5. Data Collection

During the formal study, data were collected through the Questionnaire Star platform from May 2023 to March 2024. Respondents accessed the questionnaire by scanning a QR code or clicking a link, with an IP address limited to one response each to prevent duplicate submissions. A standardized guideline explained the study's purpose and instructed participants on how to complete the questionnaire. All options, except for the “other” option, were mandatory. Demographic data such as gender, age, education, and field of work were also collected. Response times were automatically recorded by the questionnaire platform.

### 2.6. Data Analysis

Descriptive statistics were presented as frequencies and percentages for categorical variables. For continuous variables, normality was assessed using the Shapiro–Wilk test (α = 0.05); those that met the normality assumptions were summarized with means and standard deviations (SDs), whereas non-normally distributed variables were reported using medians and interquartile ranges (IQRs).

Item analysis was conducted using the critical ratio and the correlation coefficient. For the critical ratio method, respondents were classified into high-score (top 27%) and low-score (bottom 27%) groups. An independent t - test determined whether each item could significantly distinguish between these groups. Items with a critical ratio (|t|) less than 3.0 were considered to be excluded. For the correlation coefficient method, items with a corrected item-total correlation coefficient below 0.40 were considered to be excluded. Additionally, items were assessed for deletion if their removal led to increase in Cronbach's α [24].

Cronbach's α and intraclass correlation coefficient (ICC) were used to assess internal consistency and test-retest reliability, respectively. A Cronbach's α ≥ 0.70 indicated acceptable internal consistency, while ICC > 0.70 indicated acceptable time stability. The I-CVI and the S-CVI/Ave were used to evaluate content validity of the instrument. I-CVI ≥ 0.78 and S-CVI/Ave ≥ 0.90 indicate satisfactory content validity [25].

Dimensionality was evaluated using PCA with maximal rotation of variance [26]. The Kaiser Meyer Olkin (KMO) test and Bartlett's test of sphericity were performed to determine the sample fit to confirm that PCA was appropriate. Factors were extracted based on an eigenvalue > 1 and factor loading ≥ 0.40 by using PCA. Construct validity was evaluated using CFA. A robust chi-square to degrees-of-freedom ratio (*χ*^2^/*df*) < 5, Tucker–Lewis index (TLI), normed fit index (NFI), incremental fit index (IFI), comparative fit index (CFI) > 0.90, root mean square error of approximation (RMSEA), and standardized root mean squared residual (SRMR) < 0.08 indicate an acceptable data-model fit [27]. The average variance extracted (AVE) and composite reliability (CR) were used to assess convergent validity, with AVE > 0.50 and CR > 0.70 indicating good convergent validity [28]. Discriminant validity was performed using the heterogeneity-mono-trait ratio (HTMT), with a correlation matrix value < 0.85 considered excellent [29].

Simple randomization was used for sample allocation, with a random sequence generated by IBM SPSS (version 27.0). Data analysis was conducted using IBM SPSS (version 27.0), IBM AMOS (version 29.0), and Smart PLS (version 4.1.0.0). A statistically significant difference was considered when *p* < 0.05.

### 2.7. Ethical Considerations

Ethical approval was obtained from the Ethical Committee at the Nanfang Hospital, Guangzhou, China (NFEC-2023-165). Prior to data collection, all participants provided informed consent and voluntarily completed the online questionnaire. Participant information was kept confidential and anonymous.

## 3. Results

### 3.1. Characteristics of the Participants in the Validation Phase

**Figure 2:**
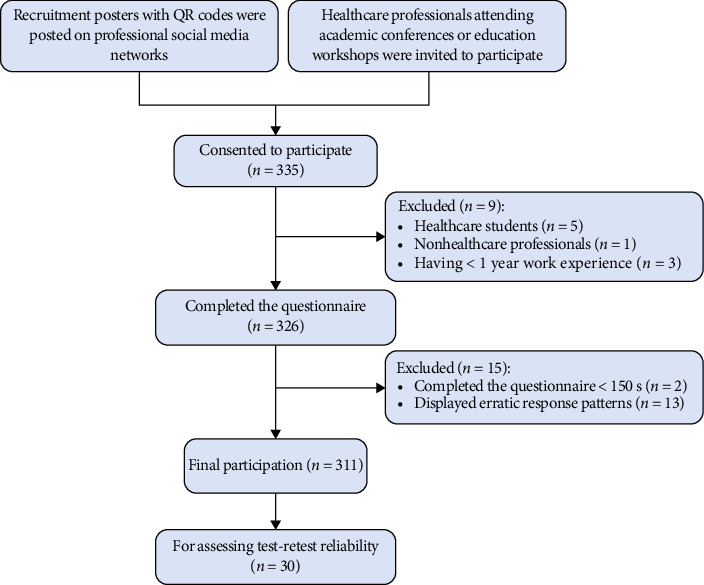
The flowchart of participants' enrollment.

A total of 335 participants were initially recruited for the study. After that, 24 participants were excluded for the following reasons: not meeting the inclusion criteria (*n* = 6), having less than 1 year work experience (*n* = 3), completing the questionnaire in less than 150 s (*n* = 2), and exhibiting erratic response patterns characterized by extreme scores (*n* = 13). Finally, 311 eligible healthcare professionals were included in the study. Of these, 284 (91.3%) participants were female, and 272 (87.4%) were nurses. The participants had an average of 14.9 ± 10.1 years of work experience, with 213 (68.4%) working directly with patients for at least five days per week.  Figure 2 displays the participant enrollment flowchart. Participant characteristics are summarized in Table 2.

### 3.2. Results of Cultural Adaption

During the expert review process, the examples in Item M4, originally “for example, prediction of competence development, communication, clear guidance, support for renewal, and participation,” were recommended to be simplified to “for example, providing clear guidance and support for education and training.” During the cognitive interviews, a participant reported that the phrase “not reluctant to” in Item C1 was difficult to understand, and it was suggested to modify to “willing to”. Specific linguistic modifications following expert review and cognitive interviews were detailed in Table S1 in Supporting Information 2.

### 3.3. Results of Item Analysis

Item analysis showed a significant difference between high-score and low-score groups. The critical ratio values for all items were above 3.0 (ranging from 17.63 to 26.70, *p* < 0.001) (Table 3), indicating excellent item discrimination. All corrected item-total correlation coefficients exceeded 0.4 (Table 3). Cronbach's α if items were deleted was acceptable (Table 3).

### 3.4. Internal Consistency and Test-Retest Reliability

Cronbach's α of the scale and its three dimensions were 0.96, 0.94, 0.92, and 0.93, respectively (Table 3), indicating excellent internal consistency. As shown in Table 3, test-retest reliability over a 2-week interval was good (ICC 0.96; 95% CI: 0.91–0.98).

### 3.5. Content Validity

To ensure content validity of the Chinese version of the DigiComInf instrument, 11 experts evaluated the relevance of the items, including 9 specialized in healthcare services and 2 in information technology. The S-CVI/Ave for the instrument was 0.97, and the I-CVI ranged from 0.91 to 1.00 (Table 3), indicating satisfactory content validity.

### 3.6. Construct Validity

**Figure 3:**
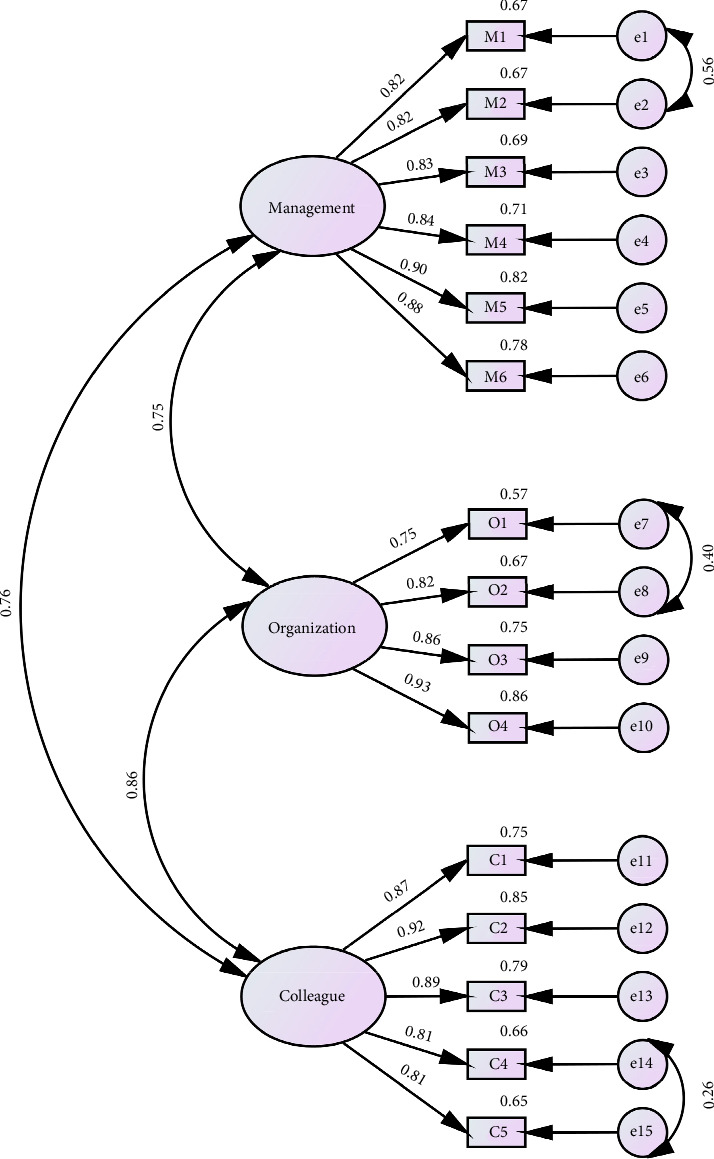
The confirmatory factor model of the Chinese version of the DigiComInf instrument (*n* = 210). Note: Management, support from management; organization, organizational practices; colleague, colleagues' adoption and influence.

For the PCA, the value of KMO (0.90) and Bartlett's test of sphericity (*χ*^2^ = 1559.18, *p* < 0.001) indicated that the data were amenable for performing factor analysis. Three factors were extracted using a criterion of an eigenvalue > 1, which explained 80.54% of the total variance. The factor loadings of items are > 0.40 (Table 3). As illustrated in Figure 3, the results of CFA supported the three-factor structure established in the original English version. The indices, including *χ*^2^/*df* (3.19), CFI (0.94), NFI (0.92), TLI (0.93), IFI (0.94), RMSEA (0.10), and SRMR (0.04), indicated an acceptable model fit. The factor loadings of items ranged from 0.75 to 0.93. The AVE values for each dimension of the instrument exceeded 0.50 (range: 0.71–0.74), with the CR values > 0.70 (range: 0.88–0.93), indicating satisfactory convergent validity. The HTMT values within the matrix were less than 0.85 (range: 0.72–0.83), indicating satisfactory divergent validity. These results collectively demonstrated satisfactory construct validity.

## 4. Discussion

We translated the DigiComInf instrument into Chinese and examined its cultural adaptation, reliability, and validity among Chinese healthcare professionals. The Chinese version of the DigiComInf proved internally consistent, time-stable, valid in content, and construct, indicating that it is a reliable and valid instrument. To our knowledge, this study represents the first effort to validate an instrument for assessing managerial, collegial, and organizational aspects associated with digital health competence among Chinese healthcare professionals.

In this study, each item was translated and back-translated following a standardized procedure [22] to ensure alignment in semantic, conceptual, and content with the original English version. We invited 11 experts from various fields, including nursing, medicine, and information technology, to test content validity of the Chinese version of the DigiComInf instrument. High content validity indices imply that the instrument provides a broad enough range of content to allow conclusions about the targeted construct. Subsequent cultural adaptation methods, such as expert review, cognitive interviewing, and pretesting, were employed to refine the linguistic expression of the instrument and ensure its clarity and comprehensibility. Issues related to semantic validation and comprehension identified in the initial Chinese version were addressed after receiving feedback from the expert review and cognitive interviewing, resulting in improvements such as clarifying complex phrase. During the expert review process, the original examples in Item M4 (“e.g., prediction of competence development, communication, clear guidance, support for renewal, and participation”) were rephrased as “for example, providing clear guidance and support for education and training,” as phrases such as “prediction of competence development” and “support for renewal” are uncommon in Chinese and therefore seem abstract. Experts suggest adopting simpler phrases. During the cognitive interviews, a participant reported difficulty understanding the phrase “not reluctant to” in Item C1 and suggested modifying it to “willing to.” This feedback highlights the importance of cultural adaptation in enhancing clarity and linguistic simplicity by avoiding double negatives and using culturally familiar examples.

Furthermore, this instrument underwent rigorous testing for internal consistency, test-retest reliability, and construct validity, exhibiting excellent internal consistency, time stability, and construct validity, resonating with the original study [20]. The types of reliability and validity examined in this study represent essential psychometric properties for a measurement instrument. Our CFA results reinforce the three-factor structure of the Chinese version, validating the PCA findings reported in the original study [20]. The DigiComInf instrument consists of three aspects associated with digital health competence that describe ‘support from management,' ‘organizational practices as part of digital competence development,' and ‘colleagues' adoption and influence.' The three aspects are widely regarded as the primary factors of digital health competence within healthcare professionals [5, 14, 30, 31]. The necessity for management support is stressed by professionals—for instance, by providing resources and opportunities for digital competence sharing, encouraging intergenerational learning and mutual learning between professionals, and fostering collaborative culture within work spaces through leadership [14]. Organizational practices and colleague influence also play a key role in developing digital health competence and building positive experiences among healthcare professionals [10, 30, 31]. For instance, to improve employees' digital health competence, healthcare organizations must ensure sufficient resources, apparatus, technological accessibility, and ongoing education [6]. Furthermore, positive appraisal and attitude of peers, particularly senior staff members, can be constructive and beneficial in identifying and enhancing challenging areas [14]. Our findings suggest that the Chinese version is a psychometrically sound instrument for measuring digital health competence, and its three-factor structure captures the interdependent socio-organizational drivers of digital health competence in healthcare settings.

The global digital transformation necessitates that healthcare professionals possess sufficient digital health competence to ensure effective implementation of digital solutions and the delivery of high-quality healthcare services [22]. Previous studies primarily focused on how individual knowledge, attitudes, and skills affect digital health competence [30, 32]. However, alongside assessing individual factors, it is essential to recognize organizational complexities that influence competence development within healthcare systems [30, 33]. In evaluating the implementation of new digital solutions in work practices and in planning educational or training activities to enhance digital health competence, a validated instrument such as the DigiComInf instrument is crucial. This instrument can measure influential factors from managerial, collegial, and organizational aspects to formulate targeted intervention strategies (e.g., enhancing organizational training and optimizing management plans), thereby promoting the development of digital health competence among healthcare professionals.

China faces regional disparities in digital health infrastructure and access, particularly in rural areas, despite the rapid growth of digital health services [34]. Most high-quality healthcare resources are concentrated in tertiary hospitals [35]. Consequently, the impact of managerial, collegial, and organizational factors on the development of healthcare professionals' digital health competence may differ across regions and healthcare institutions. When formulating strategies to enhance digital health competence of healthcare professionals in various regions or institutions, policymakers and leaders can tailor their approaches using evaluations from the Chinese version of the DigiComInf instrument. Additionally, healthcare managers can provide targeted guidance addressing individual needs, optimize personnel allocation, and cultivate a positive learning environment within the workplace community.

Furthermore, the digital health competence and its socio-organizational influencing factors might differ among healthcare roles (e.g., physicians, nurses, and allied health professionals), due to variations in scope of practice, training frameworks, and workplace responsibilities. Consequently, generic interventions might fail to address the specific technological demands and social dynamics inherent unique to each professional domain. As the DigiComInf instrument is designed for diverse healthcare professions, it serves to measure and compare influencing factors across different roles. This facilitates the integration of role-specific contextual factors into tailored strategies, thereby ensuring effective acquisition of digital health competence across multidisciplinary healthcare teams.

### 4.1. Limitations

This study presents several limitations. First, we did not evaluate the criterion validity of the instrument, due to the absence of a benchmark instrument. Second, the evaluations of the DigiComInf instrument mainly rely on subjective self-reports, which are susceptible to response biases, such as social desirability bias. Finally, the primary respondents were nurses employed within healthcare facilities and predominantly located in the southern China, which may introduce selection bias. Future research should involve a broader spectrum of healthcare professionals and diverse healthcare institutions across multiple regions.

## 5. Conclusion

This study successfully culturally adapted and validated the Chinese version of the DigiComInf instrument. Our findings suggest that the Chinese version of the DigiComInf is a reliable and valid instrument for assessing managerial, collegial, and organizational influences on digital health competence in Chinese healthcare professionals.

## 6. Implications for Nursing Management

The Chinese version of the DigiComInf instrument is ready for use in a variety of healthcare settings and among different healthcare professionals and quantifies managerial, collegial, and organizational influences on the development of digital health competence in healthcare professionals. Healthcare managers, healthcare providers, researchers, and policymakers can use it to accurately identify modifiable socio-organizational factors associated with the development of digital health competence among various healthcare professionals in various settings, thereby informing targeted interventions to enhance their digital health competence. It can also be utilized to design pertinent strategies, education programs, and policies that stimulate the advancement of digital health competence for healthcare professionals. For example, healthcare managers can conduct baseline assessments using the DigiComInf instrument to map current competence levels across professional roles. Identified gaps—for instance, low scores in “organizational practices”—can then inform targeted interventions, such as redesigning workflows to integrate digital training modules or establishing peer-led “competence circles” to foster knowledge sharing.

## Figures and Tables

**Table 1:** Demographic characteristics of experts (*n* = 11) and cognitive interviewees (*n* = 7).

No.	Sex	Age (years)	Education	Expertise	Professional title	Working experience (years)
E1	Male	34	Bachelor's degree	IT	Senior	8
E2	Female	48	Master's degree	Healthcare service	Associate chief	21
E3	Female	60	Master's degree	Healthcare service	Professor	41
E4	Female	42	Bachelor's degree	Healthcare service	Associate chief	19
E5	Female	42	Master's degree	Healthcare service	Associate chief	19
E6	Female	49	Master's degree	Healthcare service	Professor	30
E7	Female	34	Doctoral degree	Healthcare service	Senior	8
E8	Female	41	Master's degree	Healthcare service	Associate chief	20
E9	Female	39	Bachelor's degree	Healthcare service	Professor	19
E10	Female	38	Master's degree	Healthcare service	Associate chief	18
E11	Male	34	Master's degree	Information technology	Associate chief	8
P1	Female	33	Bachelor's degree	Nurse	Charge nurse	10
P2	Male	34	Bachelor's degree	Doctor	Attending physician	9
P3	Female	36	Bachelor's degree	Nurse	Charge nurse	13
P4	Male	35	Bachelor's degree	Doctor	Attending physician	10
P5	Female	32	Bachelor's degree	Nurse	Registered nurse	15
P6	Female	33	Bachelor's degree	Nurse	Charge nurse	13
P7	Male	28	Bachelor's degree	IT technician	Junior software engineer	4

*Note:* E: expert; P: cognitive interviewee.

Abbreviation: IT, information technology.

**Table 2:** Demographic characteristics of the participants in the validation phase (*n* = 311).

Variables	Value
Female, *n* (%)	284 (91.3)
Age (years), mean (SD)	37.2 (9.4)

*Education, n (%)*	
Junior vocational qualification	57 (18.3)
Bachelor's degree	197 (63.4)
Master's degree or above	57 (18.3)

*Location, n (%)*	
Southern China	261 (83.9)
Northern and Western China	50 (16.1)

*Type of organization, n (%)*	
Tertiary hospital	174 (55.9)
Secondary hospital	26 (8.4)
Community healthcare center	88 (28.3)

*Work department, n (%)*	
Inpatient	147 (47.3)
Outpatient	56 (18.0)
Administration	32 (10.3)
Operating theater	21 (6.8)
Intensive care unit	14 (4.5)
Public health	10 (3.2)
Emergency	9 (2.9)
Hemodialysis	9 (2.9)
Central sterilization and supply	4 (1.3)
Disease control and prevention	2 (0.6)
Delivery room	1 (0.3)

*Location, n (%)*	
Southern China	261 (83.9)
Northern and Western China	50 (16.1)

*Professional license, n (%)*	
Nurse	272 (87.4)
Doctor	25 (8.0)
Midwife	7 (2.3)
Others^a^	7 (2.3)

*Patient work, n (%)*	
Daily (at least 5 days a week)	213 (68.4)
Weekly (1–4 days per week)	49 (15.8)
Monthly (a few times a month)	12 (3.9)
Rarely (a few times in several months)	21 (6.8)
I do not currently work with patients	16 (5.1)
Full time, *n* (%)	311 (100.0)
Working experience (years), mean (SD)	14.9 (10.1)

^a^Physiotherapist, paramedical technician, and pharmacist.

**Table 3:** Results of item analysis, reliability, content validity, and principal component analysis for the Chinese version of the DigiComInf instrument.

Dimension	Item	Item analysis (*n* = 311)			I-CVI (*n* = 311)	Cronbach'α (*n* = 311)	ICC (95% CI) (*n* = 30)	PCA (*n* = 101)		
		Critical ratio	CITC	CID				Factor 1	Factor 2	Factor 3
Support from management						0.94	0.91 (0.81–0.96)			
	M1	17.63	0.81	0.93	1.00			0.83		
	M2	19.50	0.84	0.93	1.00			0.81		
	M3	21.27	0.83	0.93	1.00			0.76		
	M4	19.76	0.81	0.93	1.00			0.83		
	M5	25.84	0.84	0.93	0.91			0.76		
	M6	22.53	0.81	0.93	1.00			0.64		

Colleagues' adoption and influence						0.93	0.92 (0.83–0.96)			
	C1	23.47	0.80	0.92	1.00				0.71	
	C2	26.70	0.86	0.91	0.91				0.72	
	C3	25.49	0.85	0.91	0.91				0.82	
	C4	22.17	0.79	0.92	0.91				0.81	
	C5	24.44	0.82	0.92	0.91				0.84	

Organizational practices						0.92	0.92 (0.83–0.96)			
	O1	23.07	0.78	0.91	1.00					0.79
	O2	21.72	0.84	0.89	1.00					0.84
	O3	22.13	0.81	0.90	1.00					0.79
	O4	24.42	0.83	0.89	1.00					0.73

Total						0.96	0.96 (0.91–0.98)			
Rotation eigenvalue								4.32	4.26	3.50
Cumulative variance explained (%)								28.79	57.19	80.54

*Note:* CID, Cronbach'α if item deleted.

Abbreviations: CI, confidence interval; CITC, corrected item-total correlation; ICC, intraclass correlation coefficient; I-CVI, item content validity index; PCA, principal component analysis.

## Data Availability

The dataset collected in the current study is available from the corresponding author on reasonable request.

## Supporting Information

Supporting Information 1 Supporting Information 1: The Chinese version of the DigiComInf instrument.

Supporting Information 2 Supporting Information 2: Table S1—Linguistic modifications of the Chinese version of the DigiComInf instrument following an expert review and cognitive interviews. Please note that the publisher is not responsible for the content or functionality of any supporting information supplied by the authors. Any queries (other than missing content) should be directed to the corresponding author for the article.
