# Supplementary material for: Measuring Managerial, Collegial, and Organizational Aspects Associated With Digital Health Competence in Healthcare Professionals: Validation of the Chinese Version of the DigiComInf Instrument
Source: J Nurs Manag. 2025 Jun 16;2025:8854459. doi: 10.1155/jonm/8854459 (PMC12185211; doi:10.1155/jonm/8854459)
Supplement: Supporting Information 1 — The Chinese version of the DigiComInf instrument. [file 8854459.f1.pdf]

Aspects Associated with Digital Health Competence (DigiComInf) Instrument

Please tick the appropriate option after each item based on your actual situation (1=completely disagree; 2=partially disagree; 3=partially agree; 4=completely agree)

**Factor 1: Support from management**	Score			
	1	2	3	4
1. My manager's example supports the development of my digital competence				
2. My manager supports the implementation of digital solutions				
3. My manager gives feedback about the development of my digital competence				
4. My manager can lead the development of my digital competence (e.g. prediction of competence development, communication, clear guidance, support for renewal and participation)				
5. My manager supports my participation in continuing education to strengthen my digital competence				
6. Top management supports the uptake of digital solutions				
**Factor 2: Organisational practices as part of digital competence development**				
7. Education about the digital solutions used at my work has been sufficient				
8. Digital competence development is planned in my unit according to individual needs				
9. The orientation for digital solutions is conducted systematically at my work unit				
10. My organisation's practices support opportunities to develop my digital competence				
**Factor 3: Colleagues' adoption and influence**				
11. Colleagues are not reluctant to start using digital solutions at work				
12. The implementation of digital solutions has been perceived positively in my work community				
13. Colleagues are eager to develop their own work on digital solutions				
14. Colleagues do not have a negative influence on the development of my digital competence				
15. Colleagues in my work community have mainly a good level of digital competence				

卫生保健人员数字化胜任力影响因素量表

请根据实际情况勾选条目后的相应选项（1=完全不同意；2=部分不同意；3=部分同意；4=完全同意）

**维度一：管理层的支持**	选项			
	1	2	3	4
1. 上级的榜样示范有助于我发展数字化胜任力				
2. 上级支持数字化解决方案的实施				
3. 上级对我的数字化胜任力发展给予了反馈				
4. 上级可以引领我的数字化胜任力发展（如,给予清晰的指引及教育培训支持）				
5. 上级支持我参与继续教育来增强我的数字化胜任力				
6. 最高管理层支持采用数字化解决方案				
**维度二：组织实践与支撑**				
7. 与我工作相关的数字化解决方案教育已充足				
8. 我所在单位根据个人需求规划了数字化胜任力发展				
9. 我所在单位系统地进行了数字化解决方案岗前培训				
10. 我所在单位的工作实践为我提供了发展数字化胜任力的机会				
**维度三：同事的接受度与影响**				
11. 同事们愿意在工作中开始应用数字化解决方案				
12. 在我的工作社群里，数字化解决方案的实施得到了积极的评价				
13. 同事们渴望开发适用于自身工作的数字化解决方案				
14. 同事们没有对我的数字化胜任力发展产生负面影响				
15. 在我的工作社群里，同事们的数字化胜任力基本上都很好				
