# Supplementary material for: Measuring Managerial, Collegial, and Organizational Aspects Associated With Digital Health Competence in Healthcare Professionals: Validation of the Chinese Version of the DigiComInf Instrument
Source: J Nurs Manag. 2025 Jun 16;2025:8854459. doi: 10.1155/jonm/8854459 (PMC12185211; doi:10.1155/jonm/8854459)
Supplement: Supporting Information 2 — Table S1—Linguistic modifications of the Chinese version of the DigiComInf instrument following an expert review and cognitive interviews. Please note that the publisher is not responsible for the content or functionality of any supporting information supplied by the authors. Any queries (other than missing content) should be directed to the corresponding author for the article. [file 8854459.f2.docx]

**Table S1.** Linguistic modifications of the Chinese version of the DigiComInf instrument following an expert review and cognitive interviews.

| **Item** | **Initial version** | **Final version** | **Reasons for revisions** |
| --- | --- | --- | --- |
| M4 | My manager can lead the development of my digital competence (e.g. prediction of competence development, communication, clear guidance, support for renewal and participation) | My manager can lead the development of my digital competence (e.g. providing clear guidance and support for education and training) | An expert indicated that the examples in the item might be difficult to understand. It was recommended to modify to “providing clear guidance and support for education and training” |
| C1 | Colleagues are not reluctant to start using digital solutions at work | Colleagues are willing to start using digital solutions at work | A participant and an expert indicated that “not reluctant to” was difficult to understand. It was recommended to modify to “willing to.” |

M: support from management; C: colleagues’ adoption and influence.
